# Interplay of demographic variables, birth experience, and initial reactions in the prediction of symptoms of posttraumatic stress one year after giving birth

**DOI:** 10.3402/ejpt.v7.32377

**Published:** 2016-10-24

**Authors:** Julia König, Sabine Schmid, Eva Löser, Olaf Neumann, Stefan Buchholz, Ralph Kästner

**Affiliations:** 1 Lehrstuhl für Klinische und Biologische Psychologie, Katholische Universität Eichstätt-Ingolstadt, Eichstätt, Germany; 2 Klinikum Ingolstadt, Ingolstadt, Germany; 3 Refugio München, München, Germany; 4 Städtisches Klinikum München, Klinikum Schwabing, München, Germany; 5 Caritas-Krankenhaus St. Josef, Regensburg, Germany; 6 Klinik und Poliklinik für Frauenheilkunde und Geburtshilfe, München, Germany

**Keywords:** Childbirth, aetiology, predictors, posttraumatic stress, structural equation modelling

## Abstract

**Background:**

There has been increasing research on posttraumatic stress disorder (PTSD) following childbirth in the last two decades. The literature on predictors of who develops posttraumatic stress symptoms (PSS) suggests that both vulnerability and birth factors have an influence, but many studies measure predictors and outcomes simultaneously.

**Objective:**

In this context, we aimed to examine indirect and direct effects of predictors of PSS, which were measured longitudinally.

**Method:**

We assessed women within the first days (*n*=353), 6 weeks, and 12 months (*n*=183) after having given birth to a healthy infant. The first assessment included questions on demographics, pregnancy, and birth experience. The second and third assessments contained screenings for postpartum depression, PTSD, and general mental health problems, as well as assessing social support and physical well-being. We analysed our data using structural equation modelling techniques (*n*=277).

**Results:**

Our final model showed good fit and was consistent with a diathesis-stress model of PSS. Women who had used antidepressant medication in the 10 years before childbirth had higher PSS at 6 weeks, independent of birth experiences. Subjective birth experience was the early predictor with the highest total effect on later PSS. Interestingly, a probable migration background also had a small but significant effect on PSS via more episiotomies. The null results for social support may have been caused by a ceiling effect.

**Conclusions:**

Given that we measured predictors at different time points, our results lend important support to the etiological model, namely, that there is a vulnerability pathway and a stress pathway leading to PSS. PSS and other psychological measures stayed very stable between 6 weeks and 1 year postpartum, indicating that it is possible to identify women developing problems early.

**Highlights of the article:**

- Our results are consistent with a diathesis-stress model: vulnerability (antidepressant use in the previous 10 years) influenced posttraumatic stress symptoms at 6 weeks and 1 year, independently of stress (birth-related variables).
- The strongest predictor of posttraumatic stress symptoms 1 year postpartum was posttraumatic stress symptoms 6 weeks postpartum. This means that women who develop problems could be identified during routinely offered postpartum care.
- Women with a probable migration background experienced more PSS 1 year after the birth, which was an indirect effect through more episiotomies and more PSS after 6 weeks.

The issue of women developing posttraumatic stress disorder (PTSD) after giving birth has drawn increasing research attention since the first case studies (Ballard,Stanley, & Brockington, 1995) were published in the 1990s. Experts agree that in Western countries, prevalence rates of PTSD due to childbirth are between 1 and 2% (Ayers, Joseph, McKenzie-McHarg, Slade, & Wijma, 2008; McKenzie-McHarg, et al., 2015), and PTSD in the postpartum period in general (including cases due to other traumas) is above 3% in the general population and almost 16% in risk groups (Grekin & O'Hara, 2014).

In addition to the question of prevalence, the question of what leads to PTSD after childbirth is important. Childbirth is an event that usually takes place within the context of the healthcare system and can, within limits, be influenced from the outside, so theoretically policy could be informed by research on which practices surrounding childbirth are helpful or detrimental to maternal adjustment. There are two large meta-analyses examining risk factors for PTSD in general, published by Brewin, Andrews, and Valentine (2000) and Ozer, Best, Lipsey, and Weiss (2003). Both found significant but small effect sizes (all *r*<0.20) for demographic factors and medium ones (0.20<*r*<0.32) for characteristics of the traumatic event such as severity of the event and emotional reactions during it. Peritraumatic dissociation, at *r*=0.35, had the highest predictive value in the Ozer et al. (2003) study, whereas the best predictor in the Brewin et al. (2000) study was social support after the trauma (*r*=0.40). The division into predisposing factors, factors connected to the event, and factors coming into play later can also be done for birth-related posttraumatic stress symptoms (PSS; König, 2014).

In a recent German study (Vossbeck-Elsebusch, Freisfeld, & Ehring, 2014), 224 women responded to an online questionnaire battery. The authors conducted stepwise hierarchical multiple regression analyses including prenatal and birth-related variables, post-childbirth social support, and theoretically derived cognitive variables (dissociation, negative appraisals of childbirth, rumination, thought suppression, and perseverative thinking). Prenatal variables (significant: younger age and lower well-being during pregnancy) and birth-related variables (significant: peritraumatic emotions and “well-being during childbed”) together accounted for 43% of variance in PSS. Post-childbirth social support did not improve the model (44% explained variance). When the cognitive variables were included, only “well-being during childbed” remained significant, and all cognitive variables except dissociation contributed significantly, with the overall model explaining 68% of variance. These results point to a great importance of cognitive factors in PTSD; however, with the simultaneous measurement of all variables, it may reflect cognitive aspects or correlates of PTSD rather than predictors. The fact that cognitive symptoms have been included in the current edition of the *Diagnostic and Statistical Manual of Mental Disorders* (DSM-5; American Psychiatric Association [APA], 2013) definition of PTSD certainly points in that direction.

In a systematic review of predictors of PSS after childbirth, Andersen, Melvaer, Videbech, Lamont, and Joergensen (2012) developed a scoring system for the methodological quality of research studies and divided factors according to the level of evidence. The top-rated factor was subjective distress in labour, followed by obstetrical emergencies such as instrumental delivery. Intermediately rated factors included maternal prepartum and intra-/postpartum complications, infant complications, mental health difficulties, such as depression in pregnancy or before, and low perceived support from medical staff and partner. Parity, unplanned pregnancy, duration of labour, and episiotomy/perineal lacerations were among the factors with the least support.

A recent quantitative meta-analysis of 50 studies on the predictors of PTSD after childbirth (Ayers, Bond, Bertullies, & Wijma, 2016) resulted in a refined diathesis-stress model. Among the factors with the highest correlations with PSS were pre-birth vulnerability factors (depression in pregnancy, fear of childbirth, poor health or complications in pregnancy, a history of PTSD, and counselling for pregnancy or birth), as well as birth-related factors (negative subjective birth experience, operative birth [defined as assisted vaginal or caesarean], lack of support, and dissociation). PSS following childbirth showed high correlations with depression, poor coping, and stress. The authors derived a revised diathesis-stress model from their results, which contains three types of predictors (vulnerability factors, risk factors in birth, and postnatal factors). All three predictors directly influence trauma response symptoms, which in turn influence the resolution of maintenance of PTSD. There is also an influence of vulnerability factors on risk factors in birth. Many of the studies on predictors of PSS following childbirth are correlative in nature. However, current state can influence report on earlier events. Therefore, longitudinal designs are necessary to determine the importance of and possible interconnections between different predictors.

In their study of 248 women followed from 32 weeks pregnancy until 1 year postpartum, Van Son, Verkerk, Van der Hart, Komproe, and Pop (2005) report direct effects of depression during pregnancy (positive) and family history of depression (negative) on PSS at 3 months. The model also contains several aspects of the birth experience, such as social support and information from the staff, the type of delivery, pain, and perinatal dissociation. The influence of these aspects is indirect, via perinatal dissociation, and there is also an effect of depression during life on pain during delivery. These results are consistent with a diathesis-stress model. The authors, however, did not include a measure of emotional experience during the birth. Also, their measure of PSS included only intrusion and avoidance symptoms but not hyperarousal.

Garthus-Niegel, Von Soest, Vollrath, and Eberhard-Gran (2013) report on a sample of 1,499 women who were followed from gestational week 17 to 8 weeks postpartum. They measured prepartum symptoms of PTSD, fear of childbirth, symptoms of depression and anxiety as predisposing factors, divided the birth experience into objective and subjective birth factors (for the latter, a latent variable was constructed), and postpartum PSS (also a latent variable) as outcome. In this framework, the authors examined several hypotheses and found that subjective birth experience played a crucial role in the model. The relationship of fear of childbirth and postpartum PSS was fully mediated by subjective (and, to a lesser degree, objective) birth experiences, and there was a partial mediation for symptoms of depression and anxiety. At the same time, even with the birth variables in the model, three of the four predisposing factors (symptoms of PTSD, depression, and anxiety) retained their significant relationship with postpartum PSS. The greater importance of subjective compared to objective birth factors also became apparent from the fact that the relationship of objective birth factors and PSS was, to a substantial degree, mediated by subjective birth experience.

In this context, the aim of our study was to add to the literature on predictors of PSS following childbirth. We expected, in accordance with the above-mentioned studies, a negative birth experience and instrumental delivery (caesarean section, vacuum extraction, or forceps use) to be the strongest predictors for PSS 6 weeks as well as 1 year after birth. We also expected to find two separate pathways, one starting with vulnerability that existed prior to childbirth, and one at least partially independent of demographic or vulnerability factors, starting with the birth experience itself.

## Methods

### Procedure

New mothers were recruited into the study on the maternity wards of five hospitals in four Bavarian cities. Recruitment phases of 4–6 weeks were agreed upon with the hospital staff, and during those phases we tried to approach all women who had given birth to a healthy baby within the first days after delivery. Women were excluded if their babies were seriously ill (i.e., being treated in the intensive care unit). Recruitment occurred at different times in the different hospitals and took place between May 2013 and April 2014. Master-level psychology students approached new mothers in their rooms, informed them about the study, and answered any arising questions. They then collected the informed consent forms and the participants’ addresses and left the questionnaires with the women to be collected later either by the students themselves or the staff. We asked for women's mailing addresses on a separate sheet (together with informed consent), and only identified questionnaires belonging together with a code made up of letters and numbers that would not allow identification of the person. The procedure was approved by the ethics committee of the Faculty of Psychology and Educational Science, Ludwig-Maximilian University Munich, Germany. About 6 weeks later, and again 1 year after the initial assessment, more data were collected by mail. Women whose babies were seriously ill were asked to indicate this but to answer no further questions because of the ethics committee's stipulation that only women with healthy children be studied.

### Participants

**Fig. 1:**
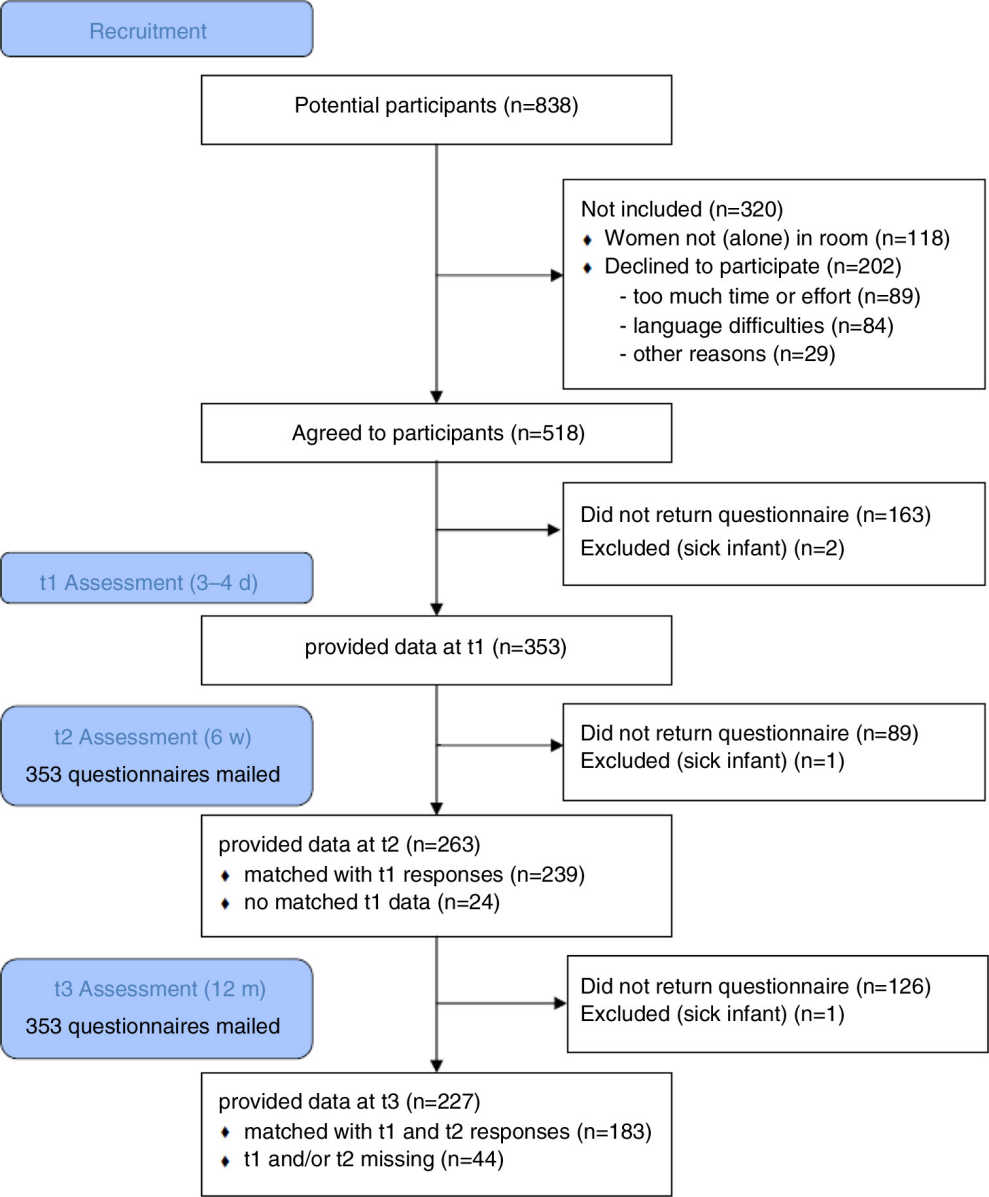
Flow chart of development of the sample.

The potential sample consisted of 838 women who gave birth during the recruitment periods in the co-operating hospitals. The flow chart in Fig. 1 illustrates the development of the sample over the three measurement time points. It has to be noted that several women (included in the flow chart as “declined to participate for other reasons”) stated explicitly that they did not want to participate because the birth had been too distressing. The response rate at the second and third assessments was rather good (75 and 64% of mailed questionnaires, respectively), but we were not able to match all received questionnaires with their counterparts from other assessments. At each time point, some participants neglected giving a code altogether, making it impossible to match their questionnaires. We matched questionnaires when only one letter or number differed, and they were probably from the same hospital. But still there was significant loss so we had usable data from all three time points from 183 women (51.8%).

### Measures

At the first measurement (t1), within days of the birth, women provided basic demographic information. As a crude measure for a migration background we asked open-ended which language participants predominantly spoke at home. We also asked for information on their pregnancy, such as whether it was planned, whether they took antidepressants during it or in the last 10 years, and whether they experienced complications such as bleeding, premature labour, or hyperemesis; or if there was a threat of a premature labour. Concerning the birth, women were asked to give the duration of labour, medical interventions (such as caesarean section, use of forceps or vacuum extraction, episiotomy), pain management (such as general anaesthesia, epidurals, and homeopathy), and birth complications (such as malpresentation, green amniotic fluid, and perineal tear). This information was all based on the women's self-report. As a measure of social support, we asked participants to indicate for several persons (partner, doctor, midwife, nurse, and friend) whether the person was present and, if so, to rate this person on a scale from “very helpful” to “very harmful”. There was space to name and rate the helpfulness of two additional people. This part of the questionnaire was checked by an experienced gynaecologist/obstetrician (last author RK) for appropriateness. Additionally, we used the German Wijma Delivery Experiences Questionnaire (WDEQ; Wijma, Wijma, & Zar, 1998). In this questionnaire, women are asked to rate their experiences during childbirth on a six-point Likert-type scale between two poles (e.g., feeling “not at all safe” to “extremely safe”). The WDEQ has good psychometric properties with a good construct validity and high reliability (Cronbach's *α*=0.93; in our sample: *α*=0.90). On the last page of the questionnaire, space was provided for any comments the women would like to share.

Questionnaires at the second (t2) and third (t3) assessments were identical and contained questions on social support, childcare, physical problems, and three self-report measures. Social support was again measured for several different people (partner, midwife, doctor, and friend) on a seven-point Likert-type scale with the anchors “very helpful” (1), “neutral” (4), and “very harmful” (7). There were six items on physical symptoms (e.g., urine incontinence) of which three were concerned with pain (e.g., pain from a caesarean section). Respondents were asked whether a symptom was present (yes/no) and, if present, to indicate the distress it caused on a seven-point Likert scale from “no distress” to “very much distress.” Furthermore, we asked women to rate their overall satisfaction with their physical state, again on a seven-point Likert scale ranging from “not at all satisfied” to “very satisfied.”

As a PTSD measure, we used the Traumatic Event Scale (TES; Wijma, 2012). There is no German version of this instrument; therefore, we translated it ourselves (with back-translation and checking with the original author). The reason for using this unvalidated measure was that the items explicitly refer to the birth experience and as we did not measure previous traumatic experiences, this seemed important to make it more likely that the reported symptoms were really due to the childbirth experience. TES items correspond closely to the DSM-IV (APA, 1994) criteria for PTSD. Criterion A1 is covered by three items, of with the first one especially has been criticised for being too easily met (“The labour/delivery was a trying experience”). We changed the wording into “… a threatening experience” in order to be closer to the DSM-IV. The original version has been shown to be reliable (Wijma, Söderquist, & Wijma, 1997: Cronbach's *α*=0.84; in our sample: *α*=0.91, *n*=235).

As PTSD after childbirth is highly comorbid with postpartum depression (McKenzie-McHarg et al., 2015), we also included the Edinburgh Postnatal Depression Scale (EPDS; Bergant, Nguyen, Heim, Ulmer, & Dapunt, 1998). This brief measure consists of 10 items covering the most important depressive symptoms during the previous 7 days and has good psychometric properties. Bergant et al. (1998) report a Cronbach's *α* of 0.81, and (for dichotomous scoring with a threshold of 9.5) a specificity of 1.0 and sensitivity of 0.96.

As a measure of general psychological distress, we included a version of the General Health Questionnaire (GHQ-12; Linden et al., 1996). Because the optimal cut-off of this measure depends very much on the sample being studied (in samples after childbirth, cut-offs between 2/3 [Saurel-Cubizolles, Romito, Lelong, & Ancel, 2000] and 6/7 [Clarke et al., 2014] have been used), we did not use the GHQ to determine caseness, but only used the sum scores (Likert scored) as a measure of general psychological distress. This measure has been found to be reliable (Cronbach's *α*=0.91) in a large German sample (Schmitz, Kruse, & Tress, 1999).

### Data analysis

As prescribed by Wijma et al. (1998), we excluded cases with more than 25% missing values on the WDEQ and replaced remaining missing values with the means of all other cases. For the TES, we replaced individual missing values with the mean score of the respective symptom cluster if possible.

After ascertaining that statistical preconditions were met, we conducted stepwise multiple regression analyses predicting TES scores at 1 year from predictors measured a few days to 6 weeks after birth. We used pairwise exclusion of cases and included the whole available sample (i.e., some women who had provided data at t2 and t3, but not t1, are included in the analyses). We did this in order to base our analyses on the largest available sample. Data collected at the two early measurement points were entered in two separate steps, and we only entered variables which correlated significantly with the t3 TES.

We used structural equation modelling to improve our understanding of the interplay of predictors. We specified an initial model including the 11 predictor variables used in the regression and with paths according to zero-order correlations between the variables. The model was then adjusted by pruning insignificant effects and variables with no significant effects on t3 TES and by using the modification indices given by the programme in order to improve model fit. For the final model, we used bootstrapping (1,000 bootstrap samples) to determine statistical significance of direct and indirect effects.

For this set of analyses, we included all participants who had provided responses at more than one assessment and imputed missing values using the “regression imputation” routine offered by SPSS AMOS. For the most part, this meant imputing data for about 10% of the sample. The one exception was t3 TES where about 20% of the sample had missing data. We considered this acceptable. The reasons for relying on imputation rather than using the cases with complete data only (*n*=179) were threefold: firstly, the correlations between t3 TES and potential predictors differed between the two samples. Secondly, women who spoke a different language at home were underrepresented in the sample with complete data (see below). Thirdly, we were concerned about the low power in a sample of 179 participants. We did *post-hoc* power analyses using an online calculator (Preacher & Coffman, 2006), which implements the routines suggested by MacCallum, Browne, and Sugawara (1996) where power is calculated from root mean square error of approximation (RMSEA), sample size, and degrees of freedom. Power was, at 0.61 for exact fit and 0.65 for close fit, rather low even in the sample including imputation.

## Results

### Demographic data, pregnancy, and birth experiences

Demographic data of the full sample at t1 are given in Table 1. At the time of the birth, women were between the 30th and 44th week of pregnancy (*M*=39.6, SD=1.7), indicating that most were delivered of term infants (31 women, or 9.0% had premature babies). One-third (33.1%) were delivered via caesarean section. The majority (*n*=277, 78.5%) of women reported that one or more medical interventions had been performed. Most women (*n*=250, 71.1%) also reported one or more birth complications, most frequently perineal tears (30.5%), followed by malpresentations (18.4%).

**Table 1:** Demographic and pre-pregnancy information in the t1 sample (*N*=353)

Variable		*M*	SD
Age		32.6	4.6
Time since last birth (months)		45.1	25.9
		*N*	%^a^
Number of previous pregnancies	0	145	41.1
	1	103	29.2
	2	51	14.4
	>2	41	7.1
Number of previous births	0	155	43.9
	1	130	36.8
	2	40	11.3
	>2	14	3.4
Relationship status	Single	5	1.4
	Relationship	98	27.8
	Married	284	70.3
Principal language at home	German	288	81.6
	German+other	43	12.2
	Other	21	5.9
Pregnancy unplanned		58	16.4
AD use in last 10 years		24	6.8
AD use in pregnancy		5	1.4

a Percentages are given of the whole sample and may not add up to 100% due to missing data. AD=antidepressant.

Sum scores on the WDEQ ranged from 16 to 131.4 points (*M*=60.9, SD=22.6). Scores of 85 or above indicate a very negative experience. In our sample, 52 women (14.7% of those with usable data) scored above this cut-off. As in other studies, reliability of the measure was good (Cronbach's *α=*0.90).

### Outcome at 6 weeks and 12 months

Before analysing the t2 and t3 data, we tested for differences in t1 responses between those with data from all assessments (*n*=183) and those who had provided data at t1 but not at t2 and/or t3 (*n*=170). Women with incomplete participation were more likely to have had unplanned pregnancies (χ^2^(1)=6.882, *p*=0.009), to speak a different language at home (χ^2^(2)=12.664, *p*=0.002), and tended to have more children (χ^2^(3)=7.235, *p*=0.065) than women with complete data. At 10%, they also had a higher rate of hyperemesis during pregnancy than their counterparts with complete data (4.4%; χ^2^(1)=4.184, *p*=0.041). There were no other significant differences at t1 between the two groups.

Sum scores of PTSD, depressive, and general symptoms, satisfaction with physical state, and social support are given in Table 2. Even though the decrease in PTSD symptoms is statistically significant, and the decrease of social support approaches significance, both changes are very small in absolute terms. Altogether, scores on the group level were very stable over the course of 1 year.

**Table 2:** Outcomes in the t3 sample (*n*≤183) 6 weeks and 12 months after childbirth

		6 weeks				12 months			Difference		
					
Variable	*n*	*M*	SD	Range	*n*	*M*	SD	Range	*t*	*df*	*p*
TES	182	22.88	5.84	17–45	183	22.00	4.69	17–39	2.568	181	0.005
EPDS	179	5.48	5.05	0–23	177	4.93	4.77	0–21	1.309	172	0.192
GHQ	182	10.20	5.35	3–28	182	10.21	4.76	4–29	0.182	180	0.856
Satis phy	182	5.55	1.37	1–7	182	5.34	1.30	1–7	1.802	181	0.073
Soc sup	183	2.23	0.59	1–4	183	2.33	0.67	1–4	−1.971	182	0.050

TES=Traumatic Event Scale; EPDS=Edinburgh Postnatal Depression Scale; GHQ=General Health Questionnaire 12; satis phy=satisfaction with physical state; soc sup=social support.

Even though there was very little pathology connected to PTSD symptoms, we analysed which variables at t1 and t2 significantly predicted symptoms of PTSD 1 year after the birth. The results of the stepwise multiple regression analyses are given in Table 3.

**Table 3:** Regression analysis of TES at 12 months

	Correlation (*n*≤208)		Model 1, *n*=193, adjusted *R* ^2^=0.179 *F*(6, 186)=7.995, *p*<0.001				Model 2, *n*=193, adjusted *R* ^2^=0.503 *F*(11, 181)=18.698, *p*<0.001			
			
Variable	*r*	*p*	*B*	SE B	β	*p*	*B*	SE B	β	*p*
Constant			17.228	1.152		<0.001	8.267	1.040		<0.001
Foreign language spoken	0.145	0.018	0.580	0.606	0.065	0.340	0.168	0.476	0.019	0.724
AD in last 10 years	0.222	0.001	4.219	1.282	0.216	**0.001**	−0.208	1.088	−0.011	0.849
Episiotomy	0.185	0.004	3.687	1.770	0.147	**0.039**	1.492	1.401	0.059	0.288
Number of medical sinterventions	0.194	0.002	0.366	0.339	0.077	0.282	0.236	0.269	0.050	0.380
General anaesthesia	0.216	0.001	3.550	1.700	0.141	**0.038**	1.731	1.372	0.069	0.209
WDEQ	0.304	<0.001	0.054	0.015	0.246	**<0.001**	0.008	0.012	0.038	0.504
TES t2	0.701	<0.001					0.483	0.070	0.532	**<0.001**
EPDS t2	0.492	<0.001					0.040	0.085	0.040	0.633
GHQ t2	0.490	<0.001					0.092	0.072	0.103	0.205
Satisfaction with physical state t2	−0.292	<0.001					0.023	0.211	0.006	0.915
Pain t2	0.359	<0.001					0.898	0.421	0.122	**0.034**

AD=antidepressant; WDEQ=Wijma Delivery Experience Questionnaire; TES=Traumatic Event Scale; EPDS=Edinburgh Postnatal Depression Scale; GHQ=General Health Questionnaire 12 item version. Significant *p* values are given in bold script.

**Fig. 2:**
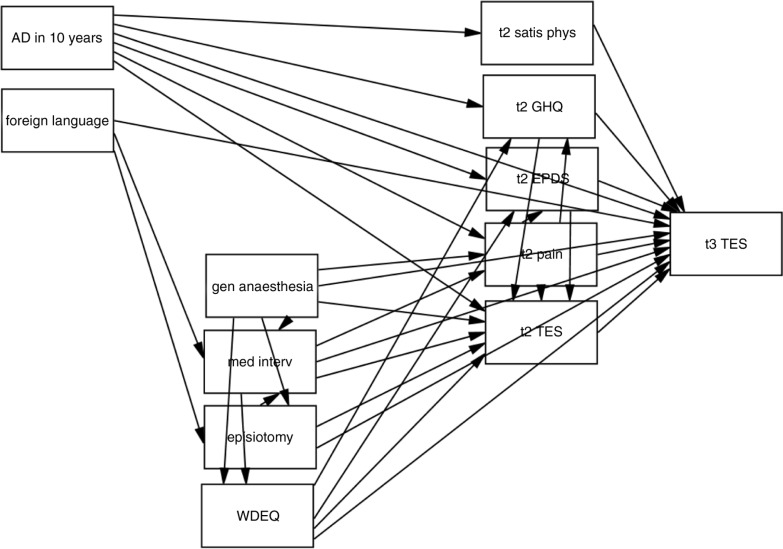
Variables and paths in the initial model. AD in 10 years=use of antidepressant in the previous 10 years; foreign language=foreign language (also) spoken at home; gen anaesthesia=general anaesthesia; med interv=number of medical interventions; WDEQ=Wijma Delivery Experience Questionnaire; satis phys=satisfaction with physical state; GHQ=General Health Questionnaire 12 item version; EPDS=Edinburgh Postpartal Depression Inventory; TES=Traumatic Event Scale; t2=assessment 6 weeks postpartum; t3=assessment 1 year postpartum.

**Fig. 3:**
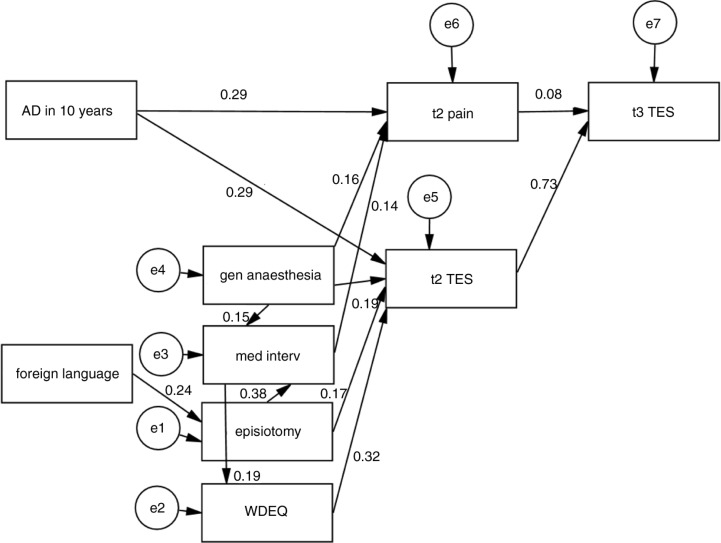
Final model. AD in 10 years=use of antidepressant in the previous 10 years; foreign language=foreign language (also) spoken at home; gen anaesthesia=general anaesthesia; med interv=number of medical interventions; WDEQ=Wijma Delivery Experience Questionnaire; TES=Traumatic Event Scale; t2=assessment 6 weeks postpartum; t3=assessment 1 year postpartum.

Although in the first model, several t1 predictors had significant effects, these disappeared when the t2 predictors were entered, suggesting the possibility of mediator effects. To test this, we used structural equation modelling. Our initial model (depicted in Fig. 2) was informed by the intercorrelations between predictors and contained the variables used in the regression. The final model (depicted in Fig. 3) had a very good fit (RMSEA=0.025, 90% CI: 0–0.057, *pclose*=0.884, GFI=0.979) and was not significant, χ^2^(23)=26.837, *p*=0.263.

All predictor variables had significant (*p*<0.05) total effects on t3 TES. Not surprisingly, given the significant regression weights, the direct effects of t2 pain and t2 TES were also significant. The same was true for the indirect effects specified in the model. In order to get an impression of the relative importance of the different predictors, we looked at their standardised total effects (STE; direct and indirect effects combined) on t3 TES. The results here are consistent with the hypothesis of two pathways—vulnerability and birth experience—leading to PSS: the strongest predictors measured at t1 were antidepressant use in the previous 10 years (STE=0.237) and WDEQ (STE=0.238). Both general anaesthesia (STE=0.163) and episiotomy (STE=0.142) had more influence than the number of medical interventions (STE=0.056). The effect of t2 TES was the highest at 0.733, whereas both speaking a different language at home (STE=0.035) and t2 pain (STE=0.084) had small, though significant, overall effects.

## Discussion

The results of the structural equation modelling analyses concur with the revised diathesis-stress model of the aetiology of PTSD after childbirth suggested by Ayers et al. (2016). Antidepressant medication during the previous 10 years had a direct effect on pain and PSS at 6 weeks which was not mediated by any of the childbirth variables (the diathesis pathway). Aspects of the birth experience (with subjective experience measured with the WDEQ as the strongest predictor) also had an effect on PSS at 6 weeks and, indirectly, PSS at 1 year (the stress pathway).

Although from a broad view, our results fit well with the literature, the same is not true for all predictors. For example, episiotomies are not mentioned in the meta-analysis by Ayers et al. (2016) and are among the lowest-rated factors in the review by Andersen et al. (2012).

The expected effect of caesarean section and instrumental delivery did not appear. Neither caesarean section nor vacuum extraction correlated with t3 TES (no woman indicated the use of forceps). However, we believe that the variable “general anaesthesia” functioned as a kind of dummy variable for more difficult caesareans. Unfortunately, we did not make the distinction between primary, secondary, and emergency caesarean sections in our questionnaire, but it is probable that there is a difference between a primary caesarean section, a secondary one that is decided on after calm deliberation, and an emergency section made necessary by immediate danger to mother and/or child. Caesareans can also be performed without general anaesthesia, and this would be the preferred method in most cases because of the advantages to early bonding. Therefore, we expect that general anaesthesia is used more often in difficult situations and emergencies. So our results are in line with the previous literature in that caesarean sections under general anaesthesia are connected to more PSS, but we were not able to find an effect of instrumental delivery (in our case, vacuum extraction). This is contrary to the results reported by Ayers et al. (2016) who found a significant medium effect for “operative birth.”

It is an interesting finding in need of replication that in our sample, having a probable migration background (that is, speaking a foreign language or both German and a foreign language at home) had a significant indirect effect on PSS at 1 year (via more episiotomies and higher PSS at 6 months). Probable migration background was also correlated with general anaesthesia, but this path was not significant in the model. It seems plausible that when complications arise during childbirth, communication problems between the mother and the staff may lead to more interventions. Therefore, this finding should probably be understood as part of the stress pathway rather than the vulnerability pathway, as speaking a different language should not be seen as a vulnerability factor *per se*. Also this variable was not an indicator of a certain ethnicity, as there was a wide range of languages named, indicating very diverse backgrounds. The fact that pain 6 weeks after childbirth played a significant role in our sample is another interesting finding. Pain in the aftermath of childbirth (rather than during) has rarely been examined in other studies. Ayers et al. (2016), for example, do not mention physical well-being after birth in their meta-analysis at all. At the same time, it has to be borne in mind that the total effects of both a probable migration background and pain at t2 are very small.

The strongest predictor for TSS 1 year postpartum was PSS at 6 weeks. In fact, none of the variables measured right after birth had a direct effect on PSS at 1 year. Also, depressive, general mental health symptoms, social support, and satisfaction with physical state, as well as PSS were very stable between the two measurements. This means that it should be possible to identify women developing mental health problems in the course of routine postpartum care.

Our study has several limitations. The most important is that we did not include a prepartum assessment. We were therefore not able to reliably distinguish between posttraumatic symptoms which may have been present before the birth and those resulting from it. However, we tried to ameliorate this problem by using the TES, which explicitly refers to the delivery experience. All information on pregnancy and birth was collected via self-report. It is possible that this introduced some bias and pulling objective data from medical records would have been preferable. As we aimed for very short questionnaires, our items measuring previous mental health difficulties (antidepressant use in the previous 10 years and during pregnancy) and migration background (language mostly spoken at home) were rather crude and consisted only of single questions. The items used to measure physical symptoms and social support were not validated. It would have been interesting to gather information about previous trauma and PTSD, dissociation, and stressors in the postpartum period. We neglected to conduct an *a priori* power analysis, and our power was rather low. It is possible that we did not find an effect for social support because of the unvalidated questionnaire. As most women were happy with the support received, this could also be an issue of too little variance. On the other hand, Vossbeck-Elsebusch et al. (2014) also did not find an effect of social support. Of course the fact that our sample was rather healthy in terms of PSS is another important limitation.

On the other hand, our study also has strengths. Especially considering its unfunded nature, it has a good sample size with any data from 384 and data from two or more assessments from 277 women. Also, we collected data at three time points after childbirth, thus circumventing the problem of confounding predictors and outcomes by assessing them at the same time as is the case for many studies. The issue of physical pain in the weeks following delivery has not been included in many of the existing studies, but it played a significant role in our sample. Interestingly, Vossbeck-Elsebusch et al. (2014) also reported that “well-being during childbed” was the only non-cognitive variable that retained a significant influence on PSS in their final model. The issue of pain in the aftermath of childbirth, as well as during, seems to merit further research in the future.

## References

[CIT0001] American Psychiatric Association (APA) (1994). Diagnostic and Statistical Manual of Mental Disorders.

[CIT0002] American Psychiatric Association (APA) (2013). Diagnostic and Statistical Manual of Mental Disorders: DSM-5.

[CIT0003] Andersen L.B, Melvaer L.B, Videbech P, Lamont R.F, Joergensen J.S (2012). Risk factors for developing post-traumatic stress disorder following childbirth: A systematic review. Acta Obstetricia et Gynecologica Scandinavica.

[CIT0004] Ayers S, Bond R, Bertullies S, Wijma K (2016). The aetiology of post-traumatic stress following childbirth: A meta-analysis and theoretical framework. Psychological Medicine.

[CIT0005] Ayers S, Joseph S, McKenzie-McHarg K, Slade P, Wijma K (2008). Post-traumatic stress disorder following childbirth: Current issues and recommendations for future research. Journal of Psychosomatic Obstetrics and Gynaecology.

[CIT0006] Ballard C.G, Stanley A.K, Brockington I.F (1995). Post-traumatic stress disorder (PTSD) after childbirth. The British Journal of Psychiatry: The Journal of Mental Science.

[CIT0007] Bergant A.M, Nguyen T, Heim K, Ulmer H, Dapunt O (1998). Deutschsprachige Fassung und Validierung der “Edinburgh postnatal depression scale” [German language version and validation of the Edinburgh postnatal depression scale]. Deutsche medizinische Wochenschrift (1946).

[CIT0008] Brewin C.R, Andrews B, Valentine J.D (2000). Meta-analysis of risk factors for posttraumatic stress disorder in trauma-exposed adults. Journal of Consulting and Clinical Psychology.

[CIT0009] Clarke K, Saville N, Shrestha B, Costello A, King M, Manandhar D, Prost A, … (2014). Predictors of psychological distress among postnatal mothers in rural Nepal: A cross-sectional community-based study. Journal of Affective Disorders.

[CIT0010] Garthus-Niegel S, Von Soest T, Vollrath M.E, Eberhard-Gran M (2013). The impact of subjective birth experiences on post-traumatic stress symptoms: A longitudinal study. Archives of Women's Mental Health.

[CIT0011] Grekin R, O'Hara M.W (2014). Prevalence and risk factors of postpartum posttraumatic stress disorder: A meta-analysis. Clinical Psychology Review.

[CIT0012] König J (2014). Posttraumatische Belastungsstörungen nach Geburt [Posttraumatic stress disorder after childbirth.]. Zeitschrift für Psychiatrie, Psychologie und Psychotherapie.

[CIT0013] Linden M, Maier W, Achberger M, Herr R, Helmchen H, Benkert O (1996). Psychische Erkrankungen und ihre Behandlung in Allgemeinarztpraxen in Deutschland. Nervenarzt.

[CIT0014] MacCallum R.C, Browne M.W, Sugawara H.M (1996). Power analysis and determination of sample size for covariance structure modeling. Psychological Methods.

[CIT0015] McKenzie-McHarg K, Ayers S, Ford E, Horsch A, Jomeen J, Sawyer A, Slade P, … (2015). Post-traumatic stress disorder following childbirth: An update of current issues and recommendations for future research. Journal of Reproductive and Infant Psychology.

[CIT0016] Ozer E.J, Best S.R, Lipsey T.L, Weiss D.S (2003). Predictors of posttraumatic stress disorder and symptoms in adults: A meta-analysis. Psychological Bulletin.

[CIT0017] Preacher K.J, Coffman D.L (2006). Computing power and effect size for RMSEA.

[CIT0018] Saurel-Cubizolles M.J, Romito P, Lelong N, Ancel P.Y (2000). Women's health after childbirth: A longitudinal study in France and Italy. British Journal of Obstetrics and Gynaecology.

[CIT0019] Schmitz N, Kruse J, Tress W (1999). Psychometric properties of the General Health Questionnaire (GHQ-12) in a German primary care sample. Acta Psychiatrica Scandinavica.

[CIT0020] Van Son M, Verkerk G, Van der Hart O, Komproe I, Pop V (2005). Prenatal depression, mode of delivery and perinatal dissociation as predictors of postpartum posttraumatic stress: An empirical study. Clinical Psychology & Psychotherapy.

[CIT0021] Vossbeck-Elsebusch A.N, Freisfeld C, Ehring T (2014). Predictors of posttraumatic stress symptoms following childbirth. BMC Psychiatry.

[CIT0022] Wijma K (2012). Traumatic event scale version B.

[CIT0023] Wijma K, Söderquist J, Wijma B (1997). Posttraumatic stress disorder after childbirth: A cross sectional study. Journal of Anxiety Disorders.

[CIT0024] Wijma K, Wijma B, Zar M (1998). Psychometric aspects of the W-DEQ; a new questionnaire for the measurement of fear of childbirth. Journal of Psychosomatic Obstetrics and Gynaecology.

